# Electrospinning and Additive Manufacturing: Adding Three-Dimensionality to Electrospun Scaffolds for Tissue Engineering

**DOI:** 10.3389/fbioe.2021.674738

**Published:** 2021-11-30

**Authors:** James A. Smith, Elisa Mele

**Affiliations:** ^1^ Department of Neurosurgery, Medical University of Graz, Graz, Austria; ^2^ Materials Department, Loughborough University, Loughborough, United Kingdom

**Keywords:** electrospininng, tissue engineering, additive manufactuing, hybrid scaffolds, nanofibers

## Abstract

The final biochemical and mechanical performance of an implant or scaffold are defined by its structure, as well as the raw materials and processing conditions used during its fabrication. Electrospinning and Additive Manufacturing (AM) are two contrasting processing technologies that have gained popularity amongst the fields of medical research i.e., tissue engineering, implant design, drug delivery. Electrospinning technology is favored for its ability to produce micro- to nanometer fibers from polymer solutions and melts, of which, the dimensions, alignment, porosity, and chemical composition are easily manipulatable to the desired application. AM, on the other hand, offers unrivalled levels of geometrical freedom, allowing highly complex components (i.e., patient-specific) to be built inexpensively within 24 hours. Hence, adopting both technologies together appears to be a progressive step in pursuit of scaffolds that better match the natural architecture of human tissues. Here, we present recent insights into the advances on hybrid scaffolds produced by combining electrospinning (melt electrospinning excluded) and AM, specifically multi-layered architectures consisting of alternating fibers and AM elements, and bioinks reinforced with fibers prior to AM. We discuss how cellular behavior (attachment, migration, and differentiation) is influenced by the co-existence of these micro- and nano-features.

## Introduction

Electrospinning is a nanofabrication technique based on the use of high electric voltages to produce polymeric fibers with micro-to nanometer diameters and distinct morphological features (Xue et al., 2019; Jain et al., 2020). A typical electrospinning process consists in ejecting an electrified polymer jet from the tip of a spinneret by applying a voltage (more than tens of kV) between the spinneret and a conductive collector. The jet is usually a polymer solution or melt, while a metallic needle connected to a syringe is used as spinneret, and a plate, disk or cylinder can be used as metallic collector (Guzman-Puyol et al., 2016). The polymer jet stretches, decreases in diameter, and solidifies before reaching the collector, where a network of solid fibers is deposited. Modifications of the standard electrospinning set-up have been proposed over the years to achieve control over fibers’ density, degree of alignment, porosity, chemical composition, and production rate. Examples include solution electrospinning, melt electrospinning, multi-jet electrospinning, needleless electrospinning, coaxial electrospinning, and near-field electrospinning (dos Santos et al., 2020).

The biomedical sector particularly benefits from electrospinning for the development of advanced systems that are relevant to tissue engineering and drug delivery (Mele, 2016; Denchai et al., 2018; Rahmati et al., 2020). One main advantage offered by this technique is flexible material selection, as a variety of biocompatible and/or biodegradable materials can easily be processed, including synthetic polymers, naturally derived polymers, and nanocomposites. In addition, the fibrous architecture generated promotes cellular activity by resembling the extracellular matrix (ECM) of native tissues. The high exposed nanofiber surface area (surface-to-volume ratio) aids in the encapsulation and release of bioactive compounds, within therapeutic limits. The high porosity control enables the mechanical performance of the fibrous mats to be tailored, in order to support cellular growth and promote the diffusion of nutrients/gases, and cell infiltration.

Strategies to further enhance the relevance of electrospun materials for tissue engineering applications have recently led to novel three-dimensional (3D) scaffolds with multi-scale hierarchical architectures (Moroni et al., 2008; Dalton et al., 2013; Balzamo et al., 2020; Chen et al., 2020a; Eom et al., 2020; Rahmati et al., 2020). These have been produced by combining electrospinning with techniques such as electro-spraying, gas foaming, phase separation, freeze-drying, additive manufacturing, leaching, and moulding.

This review focuses on recent advances in hybrid scaffold fabrication through the combination of electrospinning (melt electrospinning excluded) and additive manufacturing, and discusses how cellular behavior, such as attachment, migration, and differentiation, is influenced by the co-existence of micro- and nano-features.

## Combination of Electrospinning and Additive Manufacturing

**FIGURE 1:**
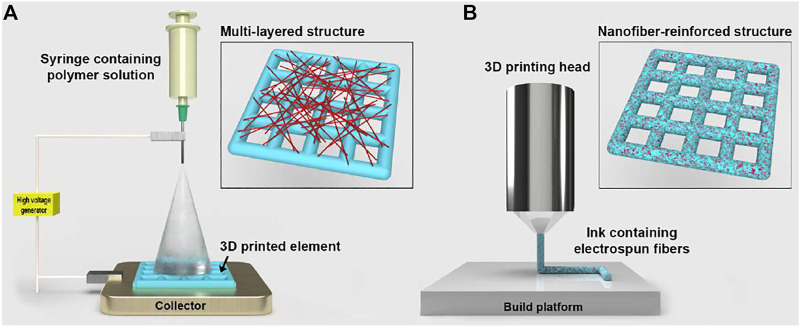
Schematic representation of the two fabrication approaches that combine electrospinning and 3D printing (elements not in scale). **(A)** Deposition of electrospun nanofibers onto one side of a 3D printed element that is placed in contact with the metallic collector of the electrospinning apparatus. Inset: 3D printed layer covered with a low-density layer of electrospun fibers. **(B)** 3D printing head depositing a polymeric ink reinforced with electrospun fibers. Inset: composite 3D printed structure where nanofibers are encapsulated within the struts.

In the literature, two main fabrication approaches that combine electrospinning and additive manufacturing have been reported so far to produce scaffolds for tissue engineering (Figure 1). These are: layer-by-layer deposition of electrospun nanofibers and 3D printed elements, referred to as multi-layered architectures; 3D printing of inks that contain electrospun nanofibers, indicated as composite inks. In addition, recent studies have explored the use of uniaxially aligned electrospun fibers to functionalise the surface of 3D printed struts to enhance cellular adhesion, proliferation and differentiation (Yeo and Kim, 2019; Yeo and Kim, 2020).

### Multi-Layered Architectures

Depositing electospun mats upon 3D printed substrates is one strategy used to develop multi-scale composite scaffolds (Moroni et al., 2008; Park et al., 2008; Mota et al., 2011; Yang et al., 2015; Naghieh et al., 2017; Rajzer et al., 2018; Yoon et al., 2019; Gao et al., 2020; Huang et al., 2020; Touré et al., 2020; Vyas et al., 2020; Yang et al., 2020; Yu et al., 2020). One seminal work proposed the manufacture of scaffolds consisting of alternating layers of randomly distributed electrospun nanofibers and 3D printed meshes (Moroni et al., 2008). It was demonstrated that these electrospun layers improved primary bovine articular chondrocyte entrapment within the scaffolds, stimulating extracellular matrix production for cartilage tissue formation. Additional research has further explored the co-existence of electrospun fibers (usually randomly distributed) and 3D printed elements within the same scaffold to influence cellular behavior (Mota et al., 2011; Yang et al., 2015). Multi-layer skin substitutes have been developed by depositing electrospun polycaprolactone (PCL) and keratin fibers on to the two surfaces of a PCL 3D printed scaffold (Choi et al., 2021), aiming at mimicking the histological structure of skin. The top electrospun layer (100 µm thick) consisted of nanofibers ( ∼ 0.7 µm average diameter) to support the growth of human immortalized keratinocytes (HaCaT); while the bottom layer (300 µm thick) was made of microfibers (∼ 1.7 µm average diameter) to enable the proliferation of normal human dermal fibroblasts (NHDF). HaCaT and NHDF cells were co-cultured onto the scaffolds for 4 days before *in-vivo* tests. Animal tests showed that the presence of keratin promoted would closure, epithelialization, proliferation of both keratinocytes and fibroblasts, and collagen deposition at the wound site. In this work, fiber’s diameter was effectively used to control cellular growth, and promote the formation of distinctive layers of keratinocytes and fibroblasts by stopping fibroblasts to infiltrate the nanofibrous layer.

**FIGURE 2:**
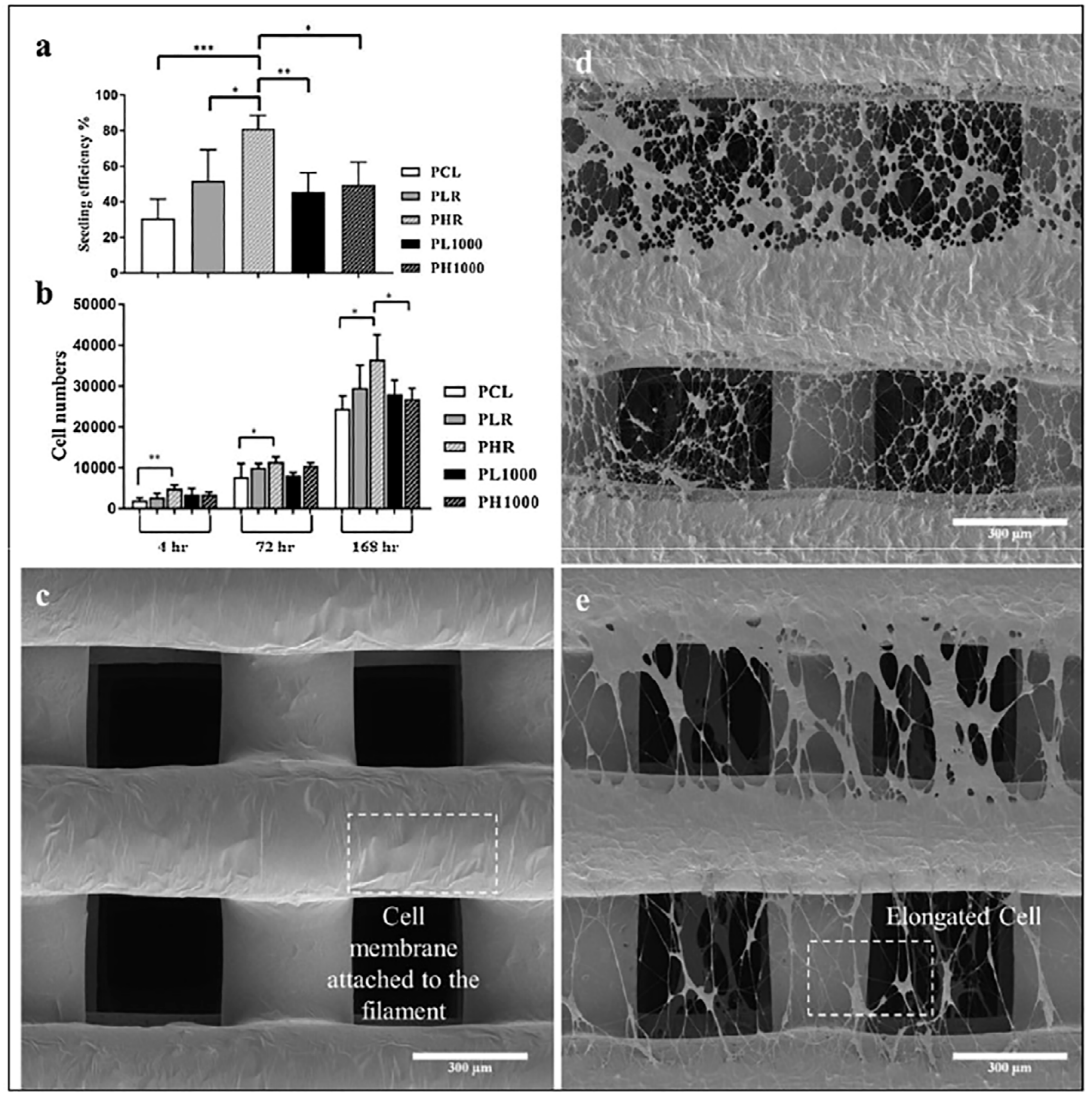
**(A)** hADSCs seeding efficiency of the following types of 3D printed PCL scaffolds (4 h of incubation): without electrospun fibers (PCL); with electrospun fibers deposited at 0 rpm for 45 s (PLR); with electrospun fibers deposited at 0 rpm for 120 s (PHR); with electrospun fibers deposited at 1,000 rpm for 120 s (PL1000); with electrospun fibers deposited at 1,000 rpm for 360 s (PH1000). **(B)** Number of hADSCs detected on the five types of scaffolds at 4, 72 and 168 h of cell culture test. Scanning electron microscopy (SEM) images of **(C)** PCL scaffold, **(D)** PHR scaffold and **(E)** PH1000 scaffold after 1 day of cell culture. The SEM images show cells attached on the structures of the scaffolds and the alignment of the cytoskeleton when aligned nanofibers are present. Reprinted from (Huang et al., 2020).

In another study, a rotating drum electrospinning system was adopted to control the density (fibers per unit area) and the degree of alignment of electrospun fibers (Huang et al., 2020). Furthermore, similar systems have been combined with screw-assisted extrusion-based additive manufacturing to produce porous PCL scaffolds for bone tissue engineering (Huang et al., 2020). PCL was firstly 3D printed (0°/90° lay down pattern, 350 µm square pores) and the resulting 3D printed element was fixed on the electrospinning collector, which was rotated at different speeds. The presence of these PCL nanofibers promoted human adipose-derived stem cell (hADSCs) attachment and influenced their osteogenic differentiation. High density electrospun mats exhibited higher cell seeding efficiencies (∼ 80%) compared to scaffolds with ( ∼ 50%) and without ( ∼ 30%) aligned nanofibers. Reductions in cell seeding efficiencies were attributed to the low porosity of the scaffolds, limiting cellular infiltration (Figure 2). The surface topography of the hybrid scaffolds greatly affected the behavior of hADSCs and aligned fibers acted as physical cues for mechano-transduction. Elongation and stretching of the cellular cytoskeleton along the direction of the fibers was observed when the hADSCs were seeded onto scaffolds with highly aligned electrospun fibers.

Recently, complex scaffolds have been manufactured to treat intervertebral disc (IVD) degeneration (Zhu et al., 2021). They were composed of three main elements: a 3D printed frame of poly (lactic acid) (PLA) to simulate the IVD structure; bundles of oriented porous nanofibres of poly (l-lactide)/octa-armed polyhedral oligomeric silsesquioxanes (PLLA/POSS-(PLLA)_8_) to mimic the annulus fibrosus (AF) structure; a gellan gum/poly (ethylene glycol) diacrylate (GG/PEGDA) double network hydrogel that encapsulated living bone marrow mesenchymal stem cells (BMSCs) to reproduce the nucleus pulposus (NP) structure. 3D printing offered great control over size and shape of the scaffolds that were tested in an animal model of IVD replacement at rat tail vertebra. The tests revealed that, after 6 months of implantation, the artificial scaffolds were effective as reimplanted autologous discs in terms of maintained disc height and production of new ECM (proteoglycans and collagen). This work demonstrates the potential of the hybrid technology (3D printing and electrospinning) for the manufacturing of personalized medical devices for IVD regeneration.

Electrospun layers can be used not only as biomimetic interface to direct cellular attachment and migration, but also as mechanical reinforcement elements, particularly when hydrogels are 3D printed (instead of thermoplastic polymers). For example, PCL electrospun mats were combined with alginate hydrogel (3D bioprinting) to create multi-layered hybrid constructs (Yoon et al., 2019).

Alginate-PCL scaffolds recorded a 4-fold increase in compressive tangential modulus ( ∼ 89 kPa with nanofibers and ∼ 23 kPa without nanofibers) at 30% strain. Furthermore, alginate-PCL scaffolds witnessed elastic recovery (without failure) up to a strain limit of 45% vs. 30% for specimens constructs without fibers. These results were credited to the elastic nature of PCL, which improved the resilience of the scaffolds. In addition, the nanofibrous layers increased the proliferation rate of NIH 3T3 fibroblasts (1.8-fold increase compared to constructs without fibers) by offering favorable perfusion conditions i.e., open porosity throughout their scaffold structure.

Electrospun nanofiber interleaves have been proposed to improve the mechanical properties of PLA 3D printed structures (He and Molnár, 2021). The nanofibrous mats were incorporated between 3D printed layers following a multi-step process. The first step was to electrospin PLA onto an aluminium foil by controlling the process time to achieve mats with the desired density/thickness. The first 3D printed layer was then deposited onto one surface of the electrospun mat. Interfacial bonding between the printed struts and the nanofibres was achieved by controlling the printing parameters and specifically the nozzle temperature (∼220°C) that reduced the viscosity of the polymer melt and facilitated adhesion. After peeling off the aluminium foil, the composite was turned upside down and a second layer was printed on the other surface of the electrospun mat. The mechanical properties of the produced 3D printed composites with nanofiber interleaves were tested in tension. It was observed a 34.3% increase in Young’s modulus for composites with a nanofiber content of 10.1 wt% if compared with neat PLA. Increases in tensile strength and elongation were also detected when 6.5 wt% and 10.1 wt% of fibres were used, indicating that the nanofibrous mats were effective in connecting adjacent printed layers and toughen the composites.

Through the combination of electrospinning techniques and flexible material selection, naturally derived polymer scaffolds that can release bioactive compounds to promote soft tissue engineering, such as cartilage regeneration, have been explored. For example, fused filament fabrication (FFF) technologies were used to manufacture poly l-lactic acid (PLLA) scaffolds, prior to the deposition of electospun gelatine and osteogenon (ossein-hydroxyapatite complex containing osteocalcin and type I collagen) nanofibers onto a single scaffold surface (Rajzer et al., 2018). Although the gelatine fibers were crosslinked with vapors of glutaraldehyde to improve their stability in aqueous solutions, the fibrous network was lost during mineralisation tests in simulated body fluid (SBF) (fusion between fibers occurred and a gel-like layer formed). After 7 days in SBF, the gelatine layer was covered by apatite crystals due to osteogenon mineralisation. In addition, *in vitro* cytotoxicity tests showed that murine fibroblasts L929 attached and proliferated onto the gelatine layer, indicating that the composite scaffolds were biocompatible.

Literature suggests that introducing electrospun nano-fibers into multi-layered scaffolds (produced by combination of electrospinning and 3D printing) has the ability to manipulate both the porosity and mechanical performance of the 3D printed constructs, as well as modulate cellular behavior (elongation and differentiation) through their intrinsic topological features. Structures made of various biocompatible and biodegradable polymers (synthetic and naturally derived) can be easily embedded within the same scaffold to obtain the desired functionality. Moreover, cell infiltration and proliferation within the scaffold can be promoted by altering density and thickness of electrospun fibrous membranes. So far, biodegradable aliphatic polyesters (PCL, PLA and PLA-based) have been primarily used for the 3D printing process, because they have a relatively low melting point, are generally inexpensive and widely available, and do not need post-processing to be crosslinked (Moetazedian et al., 2021). These polymers, however, are hydrophobic and can limit cell attachment. Future research could focus on replacing PCL and PLA with more hydrophilic materials that contain bioactive compounds to promote cell growth (proteins, growth factors, ceramic nanoparticles).

### Nanofiber-Reinforced Bio-Inks

The incorporation of electrospun nanofibers into hydrogels is discussed in the literature to create injectable composite systems that can stimulate, for example, the growth of nerve cells (Rivet et al., 2015; Omidinia-Anarkoli et al., 2017). However, so far, only a few works have reported on bio-inks that are both reinforced with electrospun nanofibers and suitable for 3D printing (Chen et al., 2019; Chen et al., 2020b). In the first paper published on this topic (inks based on electrospun nanofibers for additive manufacturing), a bio-ink containing gelatin/poly lactic-co-glycolic acid (PLGA) electrospun nanofibers was used for cartilage regeneration (Chen et al., 2019). The preparation of the composite bio-ink required multiple complex processing steps, such as the dehydration of the gelatin/PLGA mats, their homogenisation in a solvent (tert-butanol) to obtain short fibers, complete evaporation of the solvent, dispersion of the short gelatin/PLGA fibers into a viscous water solution of hyaluronic acid and polyethylene oxide (PEO). Scaffolds were then 3D printed by extrusion, before freeze-drying and crosslinking to improve their mechanical stability. This fabrication procedure enabled the formation of scaffolds with interconnected multi-scale porosity due to the 3D printed strands and the electrospun fibrous network (whose structure was preserved). In compression, gelatin/PLGA fibers determined an increase in the Young’s modulus, with scaffolds reporting values of ∼ 600 kPa; while 3D printed scaffolds containing gelatin/PLGA powder (no fibers) were characterised by Young’s modulus of ∼ 100 kPa. The ability of the 3D printed scaffolds to regenerate cartilage tissue was investigated *in vitro* with chondrocytes and *in vivo* on mice. After 6 weeks of cell culture, chondrocytes produced ECM to cover the 3D scaffolds and cartilage-like tissue was formed. Similarly, 8 weeks after animal implantation, cartilage tissue with the structure and composition to the native tissue was generated (DNA and collagen content, and Young’s modulus). These results were attributed to the porosity of the scaffolds, that allowed uniform cells infiltration and nutrients diffusion.

In follow-up studies, the bioactivity of such composite bio-inks and their regenerative potential were further enhanced by adding cartilage decellularized matrix (CDM) (Chen et al., 2020b). CDM particles were obtained from cow scapular cartilage after acellularization, freeze drying and pulverisation. They were dispersed in a hyaluronic acid solution together with gelatin/PLGA nanofibers and scaffolds were 3D printed as previously described. Articular cartilage regeneration in rabbits was observed after 12 weeks of implantation with significant deposition of collagen type II; while scaffolds without CDM showed a partially filled defect with limited collagen deposition.

The literature here discussed shows that the inclusion of electrospun nanofibers in bio-inks for additive manufacturing is an approach far more complex and time-consuming than the multi-layering methods described in *Multi-Layered Architectures* Section, because it requires additional dehydration, freeze-drying and cross-linking procedures. Nevertheless, it offers advantages in terms of obtaining scaffolds with enhanced porosity, controlled mechanical properties, and improved cellular viability. This is in line with previous research on the 3D bioprinting of hydrogels reinforced with polymeric nanofibers but not electrospun ones, including inks containing PLA nanofibers (produced by extrusion) (Narayanan et al., 2016; Kosik-Kozioł et al., 2017), and silk fibroin fibers (produced by mechanical grinding of degummed silkworm silk fibers) (Sakai et al., 2020).

## Future Outlook

The need to create functional and structurally complex scaffolds for tissue engineering has motivated the research of novel manufacturing methods that operate across different length scales. One strategy that has shown potential is the combination of electrospinning and 3D printing to enable, as discussed in this review, the production of scaffolds with multiscale porosity and hierarchical architecture. The presence of electrospun nanofibers and 3D printed micro-features within the same scaffold provides morphological and biomechanical cues that can regulate cellular behavior and promote tissue regeneration. Although a promising approach, combining these two techniques is currently time consuming and limited to research laboratories, because two separate set-ups, namely one for electrospinning and one for 3D printing, are typically required. In addition, post-processing procedures, such as freeze drying and/or crosslinking, are used to stabilise the complex structures created. Future works could focus on developing high-throughput automated systems, which integrate electrospinning and 3D printing (Mendoza-Buenrostro and Rodriguez, 2015), to achieve further control over the deposition of the nanofibers (density, thickness, and alignment degree) and increase the production rate of hybrid scaffolds.

In a recent work, a 3D printer was specifically modified to include electrospinning units (within its construct), for vascular tissue engineering applications (Fazal et al., 2021). The hybrid system can produce layered structures formed of electrospun mats and hydrogel, because it consists of one bioprinting head and two electrospinning heads (for co-electrospinning). Preliminary tests have demonstrated the fabrication of hollow tubular constructs. Gelatin methacrylate was first 3D printed around a rotating needle and photo-crosslinked; subsequently, electrospun fibers of PCL, polyvinylpyrrolidone (PVP) or polyethylene glycol (PEG) were deposited onto the external surfaces of the gelatine tubes.

The inclusion of multiple-electrospinning units within the confines of the hybrid technology could enable the fabrication and precise placement of nanofibers, constructed from different materials. This could promote localized cell-specific differentiation from Mesenchymal Stem Cells (MSCs) within the scaffold, leading to the generation of highly tunable interconnected multi-cellular/tissue systems. Such scaffolds could be used to develop sophisticated tissue models, better resembling natural tissue architectures, for future drug delivery and tissue replacement therapies. Alongside such prospects, protocols surrounding best practice e.g., sterilization methods for multi-cellular scaffolds would need to be investigated prior to implantation.
